# Generation and characterization of a PRRSV-2 DIVA marker virus by deletion of a novel epitope in the nucleocapsid protein

**DOI:** 10.1128/jvi.01259-26

**Published:** 2026-09-24

**Authors:** Jiwen Sun, Jian Wang, Kun Li, Delong Wei, Jiaoyang Li, Pu Sun, Dong Li, Pinghua Li, Yimei Cao, Lijie Tang, Zengjun Lu, Jing Zhang

**Affiliations:** 1 State Key Laboratory of Animal Disease Control and Prevention, College of Veterinary Medicine, Lanzhou University, Lanzhou Veterinary Research Institute, Chinese Academy of Agricultural Sciences 111658, Lanzhou, China; 2 College of Veterinary Medicine, Northeast Agricultural University 12430, Harbin, China; 3 Gansu Province Research Center for Basic Disciplines of Pathogen Biology, Lanzhou, China; Loyola University Chicago - Health Sciences Campus, Maywood, Illinois, USA

**Keywords:** PRRSV, nucleocapsid protein, single-B-cell antibody technology, marker vaccine, epitope

## Abstract

Porcine reproductive and respiratory syndrome virus (PRRSV) infection causes enormous economic losses to the world pig industry. Currently, there is no DIVA (differentiating infected from vaccinated animals) vaccine for PRRS available on the market. In this study, we successfully produced a porcine monoclonal antibody (C8) against PRRSV nucleocapsid (N) protein using fluorescence-based single-B-cell antibody technology. The C8 monoclonal antibody (mAb) recognizes a novel linear epitope (_92_LSDSGRISYTVE_103_) at the C terminus of genotype 2 PRRSV N protein. We developed a competitive enzyme-linked immunosorbent assay for detecting PRRSV N protein antibodies against this epitope using C8 mAb. Furthermore, we successfully rescued one PRRSV mutant, rGSWW15/N∆93. *In vitro* and *in vivo* assays showed that the mutant virus replicated efficiently and elicited a specific antibody response to PRRSV as measured by a commercial ELISA, but not to the specific epitope (_92_LSDSGRISYTVE_103_) detected by our competitive ELISA (cELISA). Our findings suggest that the recombinant virus, in combination with the cELISA diagnostic tests, enables serological differentiation between animals vaccinated with the marker virus and those infected with the wild-type virus. This study demonstrates the feasibility of developing a PRRSV DIVA vaccine through targeted modification of an N protein epitope, providing a potential strategy to facilitate PRRS control.

**IMPORTANCE:**

The development of a PRRSV DIVA vaccine is crucial for effectively managing outbreaks in swine populations. DIVA vaccines allow differentiation between vaccinated and naturally infected animals, aiding disease surveillance. In this study, we identified a novel linear epitope (92LSDSGRISYTVE103) on the genotype 2 PRRSV N protein and developed the porcine monoclonal antibody C8 targeting this epitope. A competitive ELISA (cELISA) was then created to detect PRRSV N protein antibodies specific to the C8 epitope. Additionally, we successfully generated an epitope deletion mutant, rGSWW15/N∆93. While *in vivo* assessments confirmed specific antibody responses via commercial ELISA, these antibodies did not recognize the C8-specific epitope identified in our cELISA. These findings suggest that combining the recombinant virus with cELISA diagnostic tests allows accurate differentiation between vaccinated and wild-type virus-infected animals.

## INTRODUCTION

Porcine reproductive and respiratory syndrome (PRRS) is one of the most economically burdensome swine diseases impacting the modern pig industry worldwide (1). It causes reproductive failure in sows and respiratory syndrome in pigs of all ages (2, 3). The etiologic agent, porcine reproductive and respiratory syndrome virus (PRRSV), emerged in Europe in the late 1980s and in North America in the early 1990s (4, 5). PRRSV is an enveloped, positive-sense, single-stranded RNA virus classified within the order Nidovirales, family Arteriviridae (6). The International Committee on Taxonomy of Viruses (ICTV) has revised the taxonomic classification of PRRSV, recognizing PRRSV-1 and PRRSV-2 as two separate species because of their substantial genetic divergence. According to the updated nomenclature, PRRSV-1 is classified as Betaarterivirus europensis, whereas PRRSV-2 is assigned to Betaarterivirus americense. The PRRSV genome is approximately 15 kb in length and contains at least 11 open reading frames (ORFs): ORF1a, ORF1aTF, ORF1b, ORF2a, ORF2b, ORF3 to ORF7, and ORF5a. ORF1a and ORF1b encode two polyproteins, pp1a and pp1b, which are cleaved into at least 16 non-structure proteins (nsp1α, nsp1β, nsp2, nsp2N, nsp2TF, nsp3-nsp6, nsp7α, nsp7β, and nsp8–nsp12) (7–9). The viral genome encodes eight structural proteins, including membrane proteins (GP2a, GP2b[E], GP3, GP4, GP5a, GP5, and M) and N protein (9).

At present, vaccination is one of the most effective methods to control PRRS. Modified live virus vaccines and killed vaccines are available on the market. However, pigs immunized with modified live vaccines are serologically indistinguishable from naturally infected pigs. A genetic marker vaccine would be valuable in the control and eradication of PRRS by enabling the differentiation of infected from vaccinated animals (DIVA). For some RNA and DNA viruses, live-attenuated vaccines have been developed using the insertion of a foreign protein (epitope) to produce a positive marker or the deletion of an immunogenic protein (epitope) to produce a negative marker (10, 11). Antibodies raised against the exogenous or viral protein (epitope) are utilized to distinguish immunized animals from naturally infected ones. Previously, marker vaccines for foot-and-mouth disease virus (FMDV) were developed by deleting or modifying residues in non-structural proteins 3A, 3B, and 3D. These vaccines enable the differentiation between animals vaccinated with the marker vaccine and those infected with the wild-type virus (12–14). As a small RNA virus, PRRSV encodes only a limited number of proteins with crucial functions, making it challenging to delete a viral protein entirely (15–17). Thus, an alternative approach to negative markers for PRRSV involves identifying immunogenic non-essential epitopes within viral proteins.

An ideal marker should have two fundamental characteristics. (i) The candidate marker should be immunodominant, thereby recognized by the majority of the population infected with the wild-type virus (18–20). (ii) Its deletion from the vaccine virus genome would not affect the viability and immunogenicity of the vaccine. The N protein is the most abundant protein of the PRRS virus, forming the core of the nucleocapsid complexed with viral genomic RNA (21). The PRRSV N protein is highly immunogenic, with relatively conserved amino acid sequences across genotypes. Antibodies against the N protein appear early at 7–9 days post infection, although these antibodies typically lack neutralizing activity (22, 23). Given that the majority of antibodies generated during PRRSV infection in swine sera target the N protein, which harbors highly conserved antigenic determinants, the N protein has been utilized for detecting PRRSV-specific antibodies and for the development of diagnostic reagents (24). Several commercially available ELISAs utilize recombinant PRRSV nucleocapsid proteins as antigens (25, 26). Conserved immunodominant epitopes on the N protein can be considered as candidate markers for the DIVA vaccine.

The single B cell antibody technique is a novel methodology to identify antigen-specific B cells. It includes several steps: (i) identification and isolation of single B cells; (ii) amplification, sequencing, and cloning of single-cell Ig gene transcripts; and (iii) reactivity screening of antibodies. The single B cell antibody technique, which relies on the direct amplification of VH and VL sequences from single B cells, is an efficient strategy for the development of monoclonal antibodies (mAbs). This approach preserves the original VH and VL pairing as found in host B cells (27). Previously, our laboratory established a platform for screening single B-cell antibodies against FMDV, identified several neutralizing antibodies of porcine and bovine origin, and developed ELISA methods for the detection of FMDV-specific neutralizing antibodies (28–30).

In this study, we established a system to isolate PRRSV-specific antibodies from porcine and successfully obtained nine antibodies against the N protein. We identified a novel linear epitope (_92_LSDSGRISYTVE_103_) at the C terminus of genotype 2 PRRSV. Additionally, we developed a competitive ELISA method using the C8 mAb against PRRSV as the detection antibody. We successfully rescued a PRRSV mutant, named rGSWW15/N∆93, derived from a genotype 2 strain of PRRSV. The mutant carries a point deletion at serine 93 of the N protein. To evaluate its potential use as a vaccine against PRRSV infection, the *in vitro* replication efficiency and *in vivo* biological properties of the mutant virus were characterized.

## RESULTS

### Screening for optimal pig donors for B cell sorting

**Fig 1:**
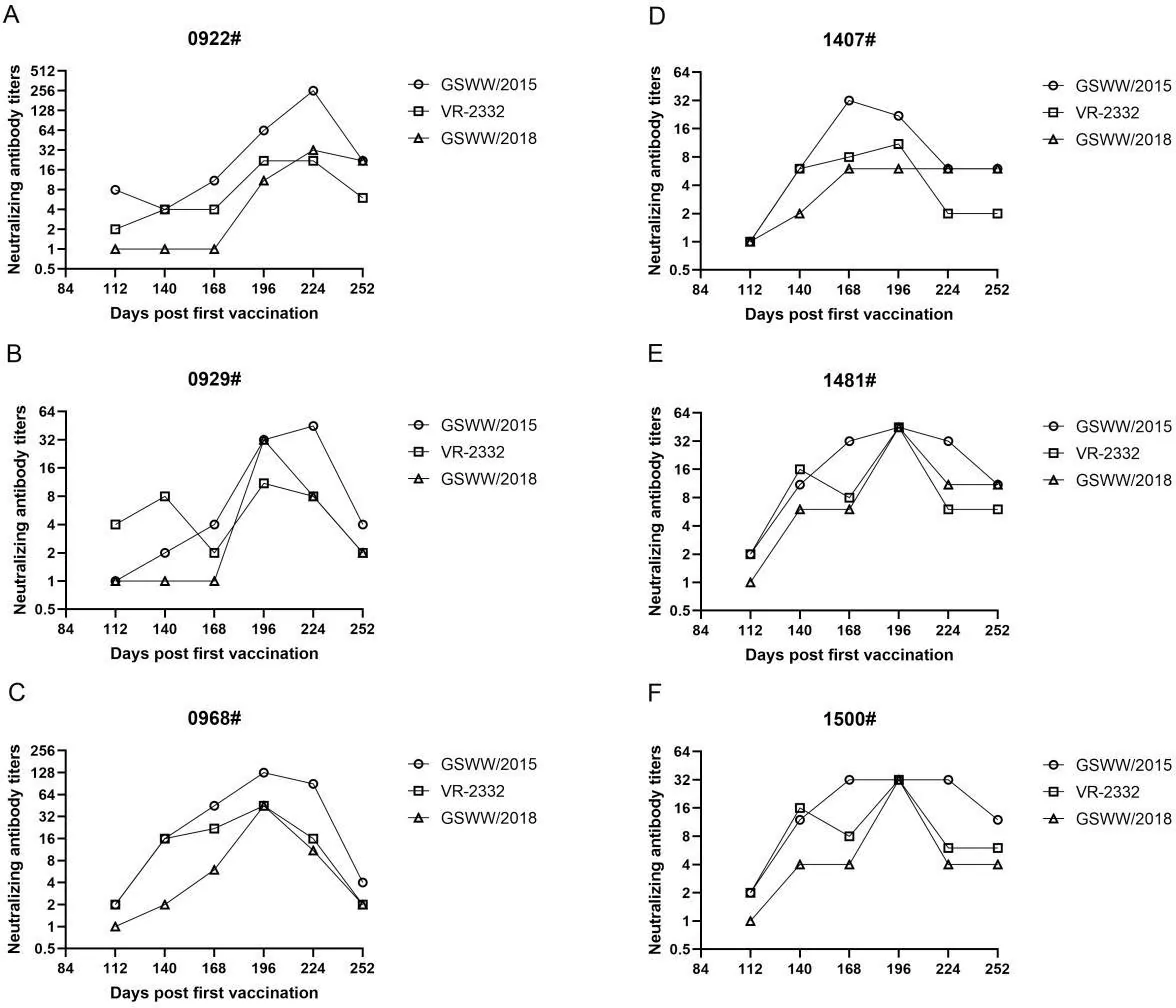
Three PRRSV-negative Juema pigs (0922#, 0929#, and 0968#) (**A–C**) and Landrace pigs (1407#, 1481#, and 1500#) (**D–F**) were inoculated intranasally or intramuscularly with various PRRSV strains several times. Serum samples from vaccinated pigs were examined for neutralizing activity against different PRRSV strains, including VR2332, GSWW/2015, and GSWW/2018, at day 112, day 140, day 168, day 196, day 224, and day 252.

To select an optimal pig donor for single B cell sorting, three PRRSV-negative Landrace and Juema pigs were inoculated intranasally and intramuscularly with various PRRSV strains several times. Serum samples from vaccinated pigs were examined for neutralizing activity against different PRRSV strains, including VR2332, GSWW/2015 (GenBank accession number: KX767091.1), and GSWW/2018 (GenBank accession number: OP764591). Among the tested pigs, only Juema pig 0922# exhibited a high level of neutralizing antibodies against GSWW/2015 (Fig. 1). Consequently, peripheral blood mononuclear cells (PBMCs) from this pig were utilized for single B cell sorting.

### Purification of PRRSV virions

PRRS virus particles were purified using sucrose density gradient centrifugation and visualized under electron microscopy following negative staining. The results are illustrated in Fig. 3G. Complete PRRS virus particles with capsule membrane wrapping could be clearly observed. The virus particles exhibited a spherical shape with varying sizes. The majority of viral particles ranged between 50 and 80 nm in diameter, with only a few particles exceeding 120 nm. This variation could be attributed to the fusion event between the viral vesicle membrane and the cell or organelle membrane during virus maturation, leading to an increased particle diameter.

### Screening of PRRSV-specific mAbs using single-B cell antibody technology

**Fig 2:**
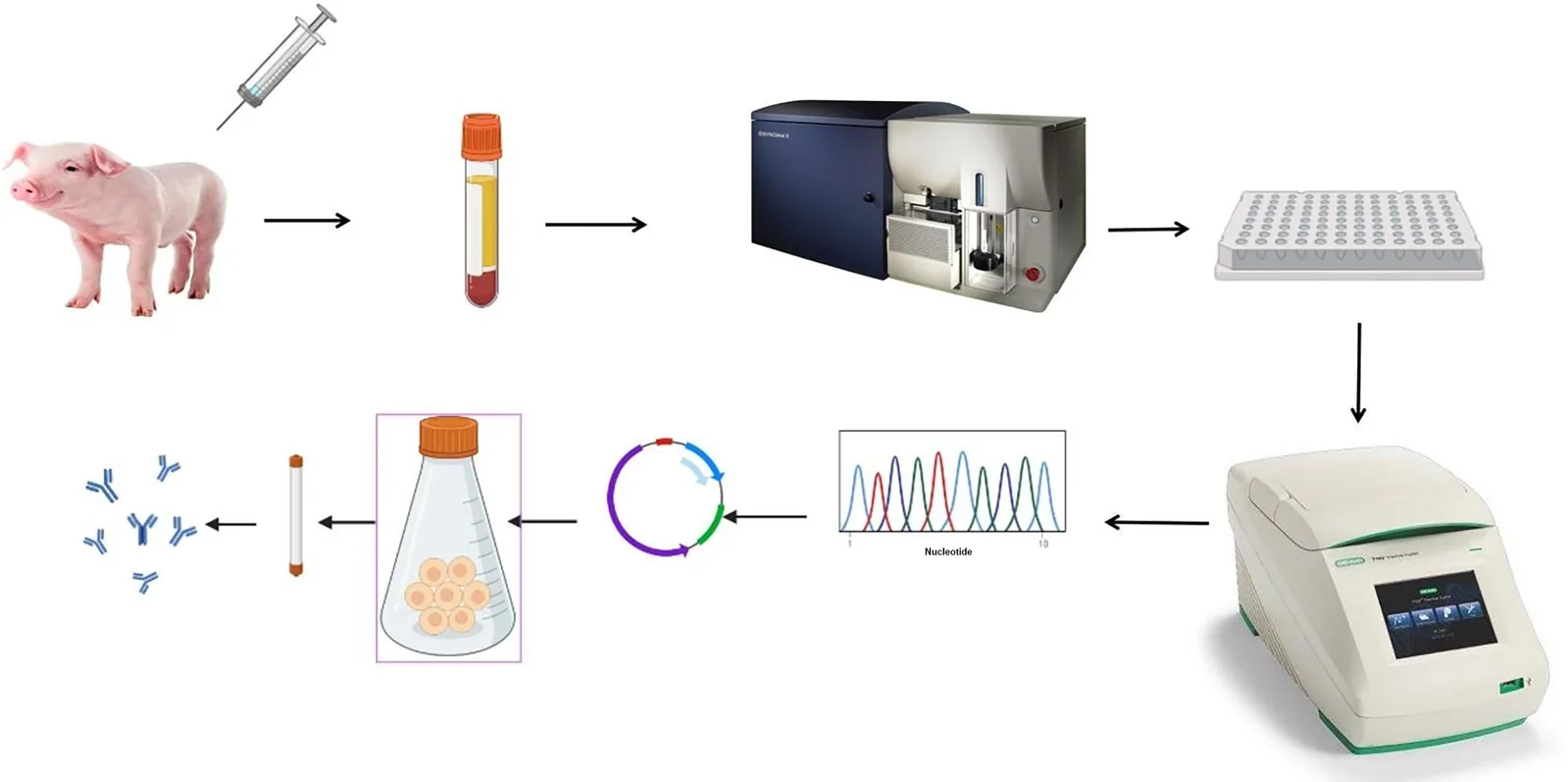
Workflow of porcine mAb production using single B cell antibody technique. Pigs were sequentially immunized with different PRRSV strains, and peripheral blood was collected to isolate PBMCs. The isolated PBMCs were stained with anti-pig IgG FITC, anti-pig IgM PE, and anti-biotin APC antibodies. The single cells were sorted into 96-well plates with lysis buffer and immediately reverse-transcribed into cDNA. The paired VH and VL sequences were amplified by nested PCR and sequenced. The variable regions were codon optimized and cloned into the pcDNA3.4 expression cassette containing porcine IgG constant region coding sequences. The recombinant mAbs were expressed in CHO-S and purified by affinity and molecular sieve chromatography.

**Fig 3:**
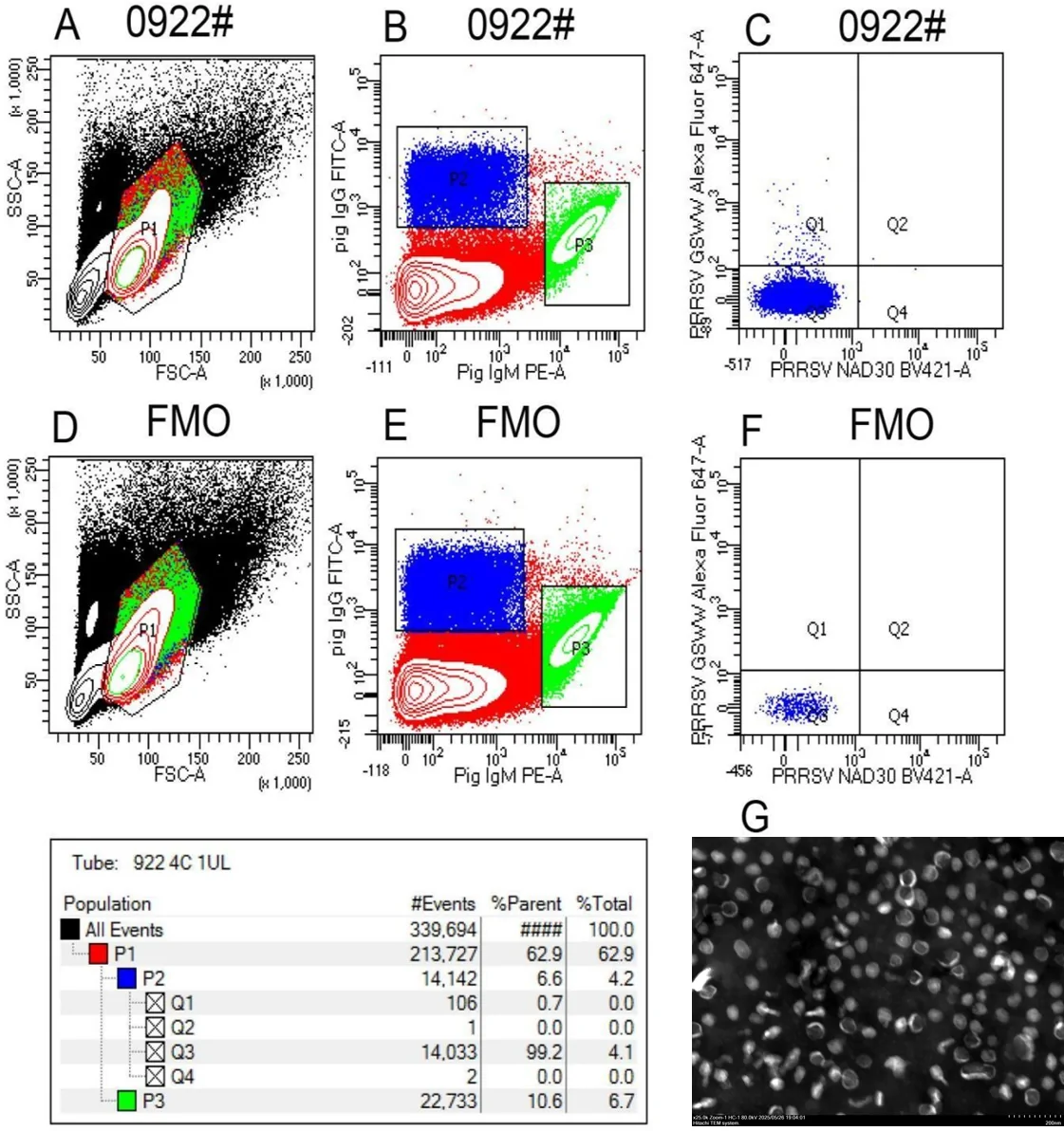
Confirmation of the proportion of PRRSV-specific plasmablasts in the peripheral blood by flow cytometry. Porcine PBMCs from pig 0922# were analyzed by three-color flow cytometry, and (**A and D**) gate 1 was selected to exclude cell debris with lower values of SSC-A and FSC-A. (**B and E**) The IgM− and IgG+ B cells were gated to check the distribution of GSWW/2015 (HP-PRRSV) and GSWW/2018 (NADC30-like) specific cells in the presence (**C**) or absence (**F**) of PRRSV antigen. One million PBMCs were collected per sample. (**G**) Electron microscopy of purified PRRSV virions.

**Fig 4:**
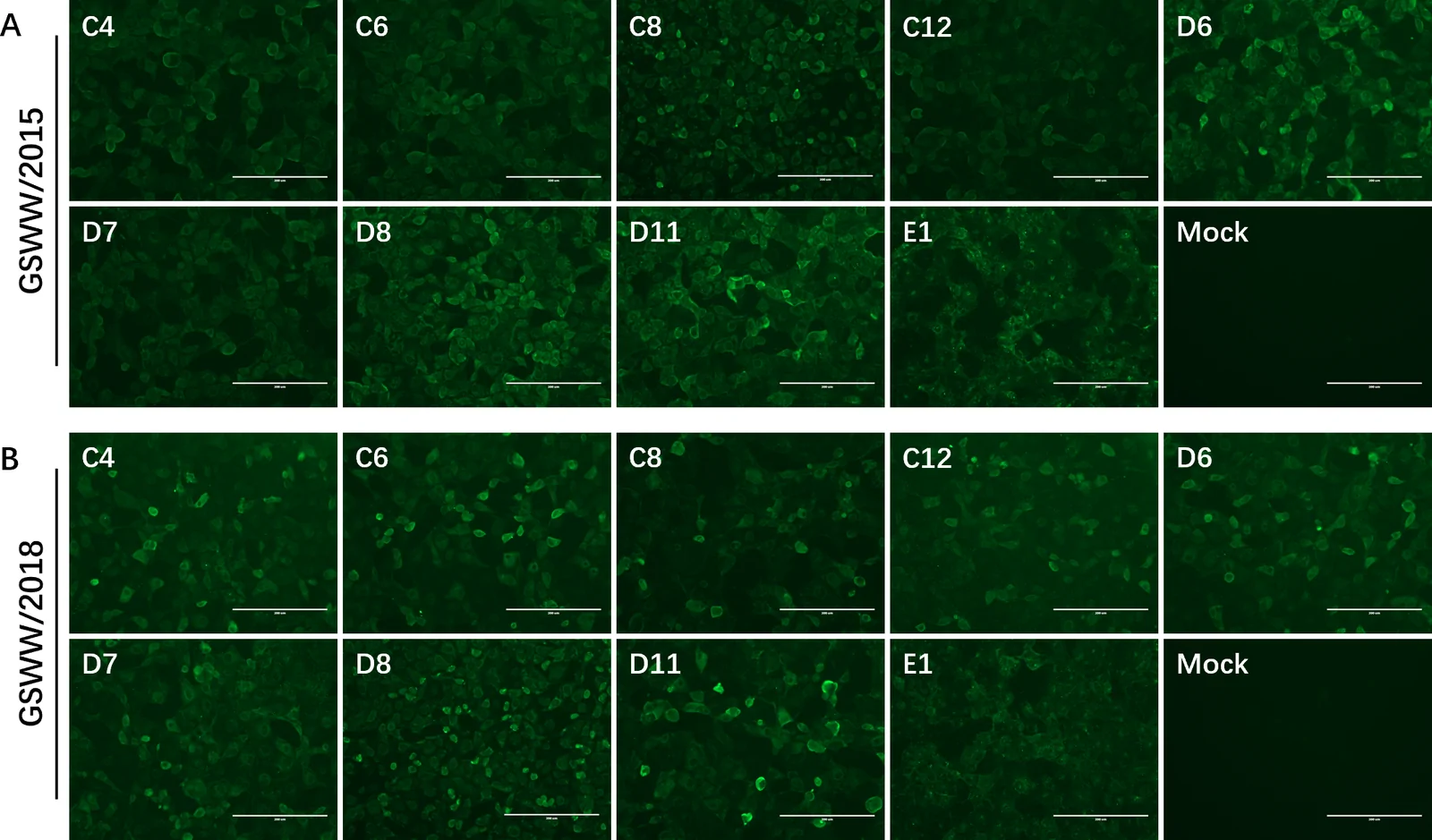
Characterization of PRRSV-specific mAbs. The reactivity of porcine mAbs was verified by IFA. The IFA was performed using MARC-145 cells infected with (**A**) GSWW/2015 strain and (**B**) GSWW/2018 strain, and the working concentration of the tested porcine mAbs was 2 µg/mL.

**Fig 5:**
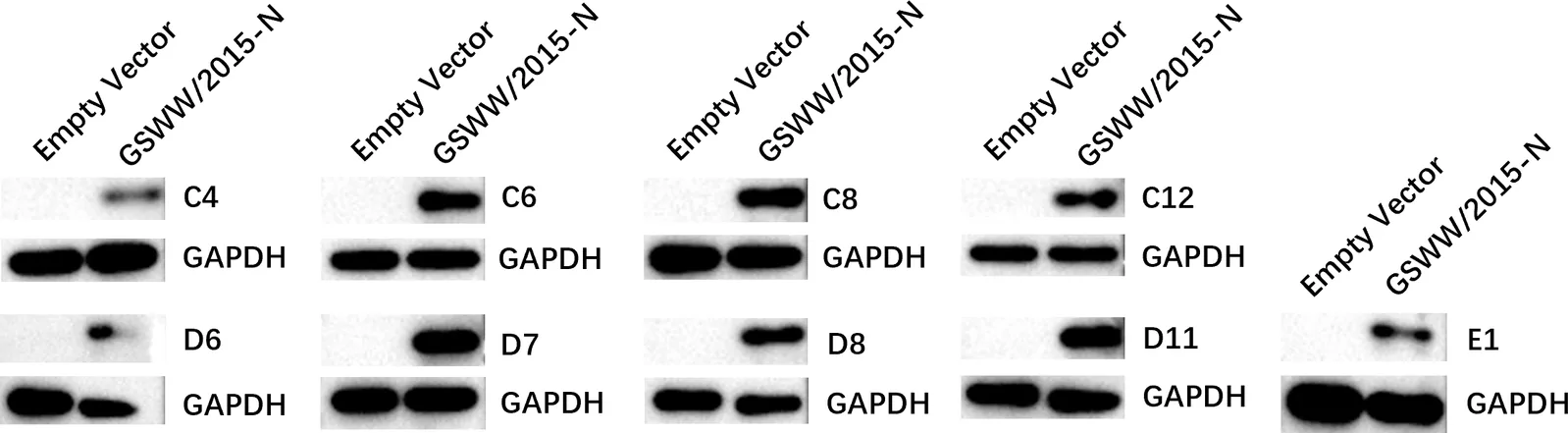
The reactivity of the porcine mAbs was verified by western blot. HEK293T cells were transfected with the N protein expression vector, and western blot was performed to detect the reactivity of the mAbs against the N protein.

The strategy employed in our study involved antigen-specific single B cell sorting and mAb production to isolate PRRSV-specific antibody-producing B cells (Fig. 2). Initially, we sorted a total of 3 × 10^6^ PBMCs for PRRSV^+^/IgG^+^/IgM events. The fluorescence-activated cell sorting (FACS) results revealed that the PRRSV-specific B cells constituted less than 0.1% of the total PBMCs in the pig (Fig. 3). Following single-cell lysis and cDNA synthesis, we employed nested PCR to amplify the variable region of the heavy and light chain genes from sorted PRRSV-specific single B cells. Subsequently, PCR products underwent analysis via agarose gel electrophoresis. Positive clones exhibited signals of approximately 500 bp for the VH and Vκ/Vλ genes. A total of 18 Ig genes of paired heavy and light chains had been successfully cloned. The antibodies were expressed in CHO-S cells in complete IgG format, and supernatants were collected for purification. The reactivity of these engineered porcine monoclonal antibodies was tested by western blot and immunofluorescence assay (IFA). IFA results showed that nine clones of the porcine mAbs (C4, C6, C8, C12, D6, D7, D8, D11, and E1) reacted with PRRSV-infected cells (Fig. 4). Western blot analysis of ectopically expressed all PRRSV proteins showed that these nine antibodies recognize the N protein of PRRSV GSWW/2015 strain (Fig. 5).

### Identification of a novel linear epitope at the C terminus of N protein

**Fig 6:**
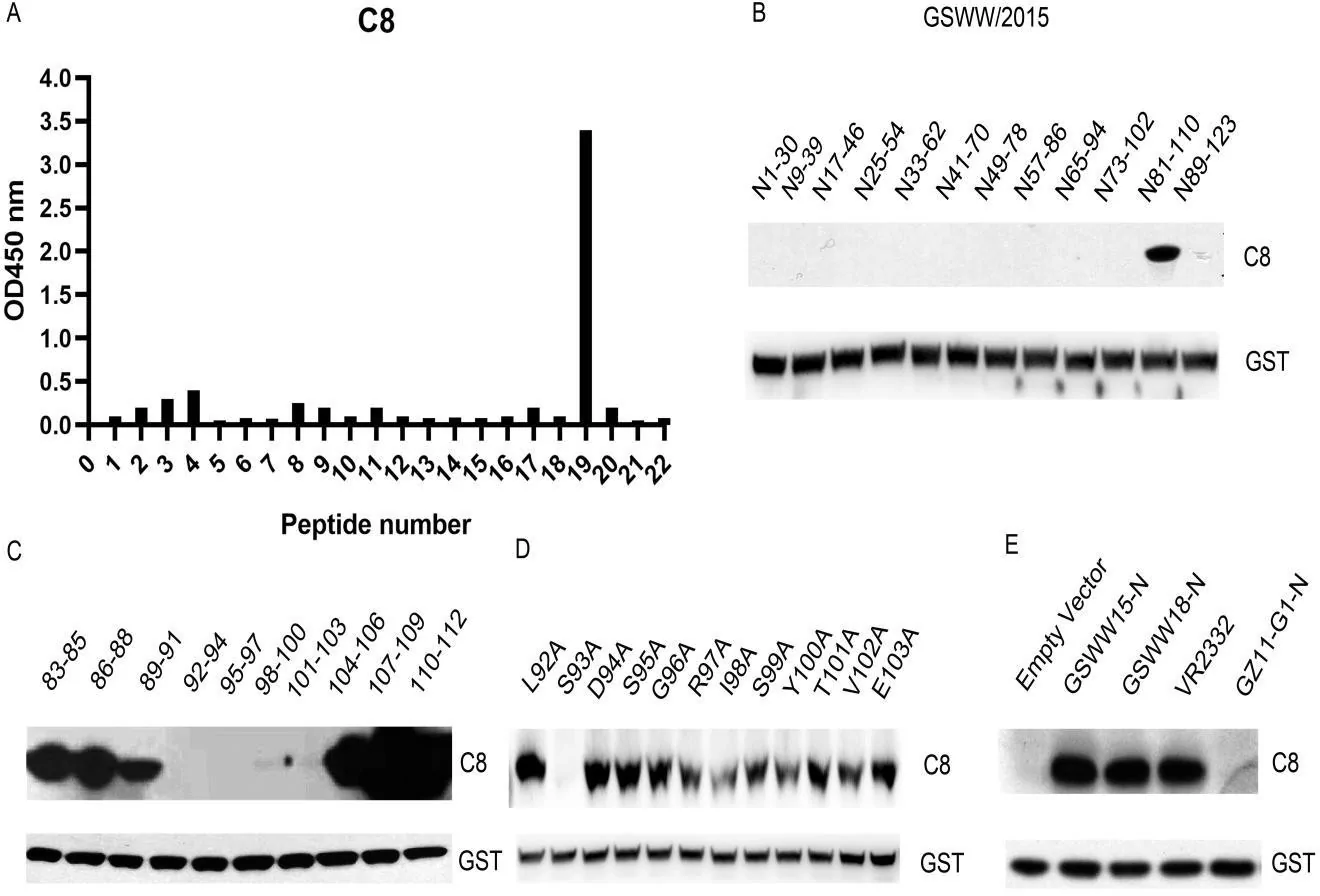
Determination of the epitope of C8. (**A**) The reactivity of C8 mAb against 22 overlapping peptides, which cover the full length of PRRSV GSWW/2018 N protein sequence, was determined by peptide ELISA. (**B**) The reactivity of C8 mAb against the truncated N proteins fused with GST was detected by western blot. (**C**) The reactivity of C8 mAb against the GST fusion proteins with three-alanine substitutions spanning the 81–110 aa was detected by western blot. (**D**) The reactivity of C8 mAb against GST fusion proteins containing single-point mutations from 92 to 103 aa of the N protein was detected by western blot. (**E**) HEK293T cells were transfected with different strains of PRRSV N protein expression vector, and western blot was performed to detect the reactivity of the mAbs against N proteins.

Peptide ELISA was utilized to identify the epitopes recognized by these mAbs. A total of 22 overlapping peptides covering the full length of PRRSV GSWW/2015 N protein sequence were coated onto plates, and their reactivity with monoclonal antibodies was determined. The results showed that all the monoclonal antibodies only reacted with peptide number 19, which contains amino acids from 91 to 105 of GSWW/2015 N protein (Fig. 6A; Fig. S1). We chose C8 for detailed study. To further validate the epitope recognized by C8, we expressed 12 truncated GST fusion proteins spanning the entire type 2 PRRSV N protein. Western blot results demonstrated that C8 reacted with the truncated N proteins containing amino acids 81–110 (Fig. 6B). To illustrate the minimal sites of C8 mAb binding, GST fusion proteins with three-alanine substitutions spanning amino acids 81–110 were expressed in *Escherichia coli* and probed. The western blot results demonstrated that introducing three-point mutations from amino acids 92 to 103 completely abolished the reactivity of C8 with the fusion proteins (Fig. 6C). Subsequently, GST fusion proteins containing single-point mutations from amino acids 92 to 103 of the N protein were generated to identify the critical amino acid for antibody recognition. The results showed that mutating serine to alanine at position 93 diminished the reactivity of C8 (Fig. 6D). These results indicate that _92_LSDSGRISYTVE_103_ is the epitope that C8 recognizes and 93S is the critical amino acid.

To explore the reactivity of C8 with the N protein of different genotypes of PRRSV, we ectopically expressed the N protein of different PRRSV strains GSWW/2015 (genotype 2, HP-PRRSV), GSWW/2018 (genotype 2, NADC30-like), VR2332 (genotype 2), and GZ11-G1 (genotype 1) in HEK293T cells and analyzed by western blot. The results showed that the C8 antibody could react with the N protein of the genotype 2 PRRSV, but not with the N protein of the genotype 1 PRRSV (Fig. 6E). Sequence alignment results showed that the epitope is conserved in the genotype 2 PRRSV but not in the genotype 1 PRRSV (Fig. S1). These results suggest that this epitope is specific in the genotype 2 PRRSV.

### Serological detection of PRRSV antibodies in clinical serum samples

A competitive ELISA based on the C8 monoclonal antibody was established to detect antibodies targeting the PRRSV N protein in pig sera. A total of 1,169 pig serum samples were collected from different regions and pig production systems in Gansu Province, China (Table 1). PRRSV-2 antibodies were detected in 716 samples, corresponding to an overall antibody positivity rate of 61.2% (716/1,169). The results demonstrated a high level of PRRSV-2 exposure among pig herds in Gansu Province. Based on vaccination status and sample origin, the samples were divided into three groups: the vaccinated farms, the non-vaccinated farms and the field samples. The antibody positivity rates in the vaccinated farms ranged from 69.0% to 92.6%, with an average of 80.1%. The antibody positivity rates in the non-vaccinated farms ranged from 42.5% to 100%, with an average of 69.5%. In contrast, the field samples exhibited relatively lower positivity rates, ranged from 0% to 24.1%, with an average of 15.6%.

**TABLE 1:** Serological evaluation of clinical pig serum samples

Type of pig farm	Serum source	No. of sera	No. of positive sera	Positivity rate (%)	Total no. of sera	Total no. of positive sera	Avg positivity rate (%)
Vaccinated farms	1	97	74	76.3	457	366	80.1
	2	82	62	75.6			
	3	56	51	91.1			
	4	28	23	82.1			
	5	100	69	69.0			
	6	94	87	92.6			
Non-vaccinated farms	7	108	97	89.8	443	308	69.5
	8	20	20	100			
	9	87	37	42.5			
	10	86	43	50.0			
	11	82	53	64.6			
	12	60	58	96.7			
Field samples	13	28	0	0	269	42	15.6
	14	60	12	20.0			
	15	94	9	9.6			
	16	87	21	24.1			

### Recovery of a mutant PRRSV strain lacking the epitope that C8 recognizes

**Fig 7:**
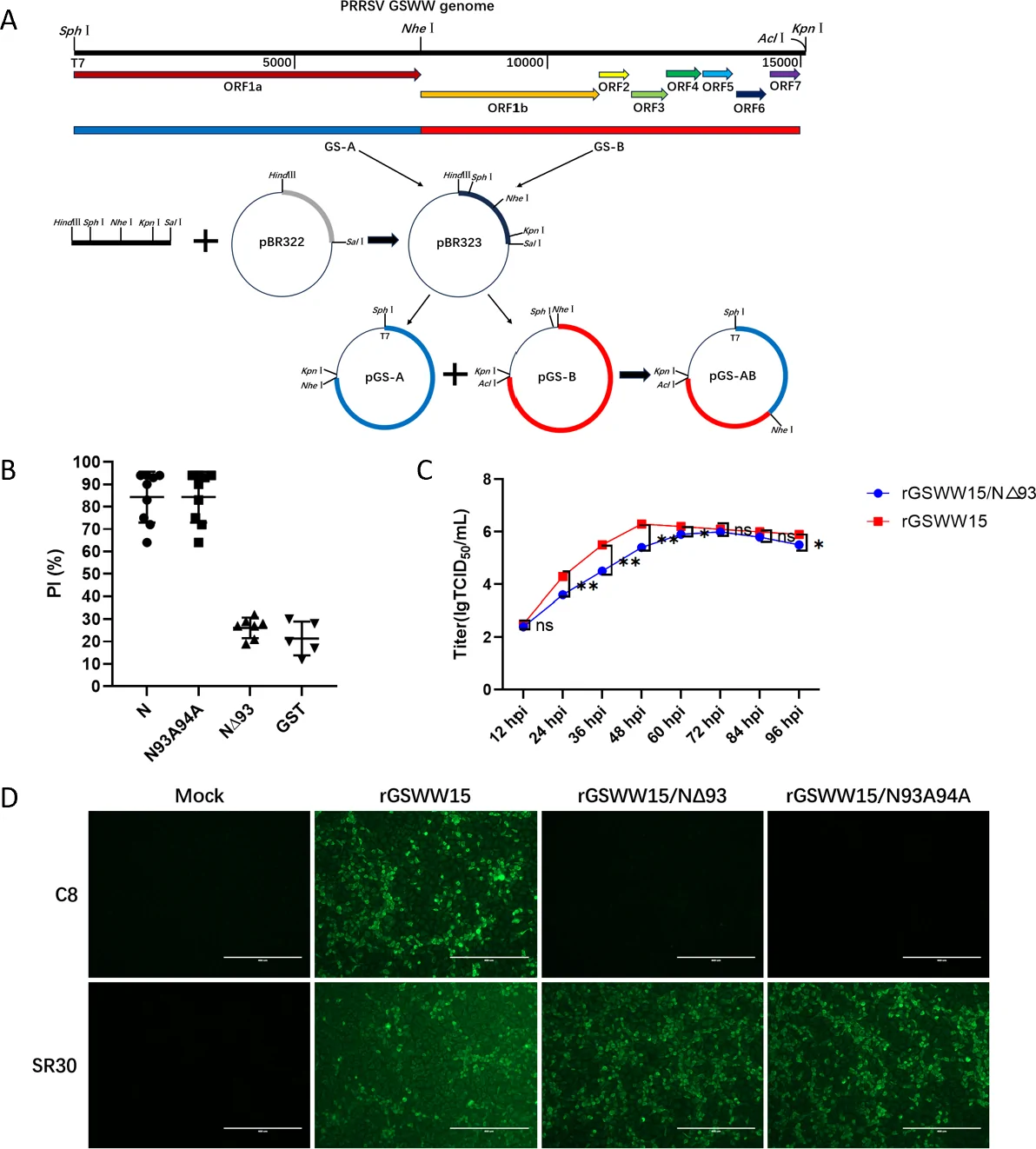
Rescue of the mutant marker viruses. (**A**) Schematic diagram of the construction of the full-length infectious clone pGS-AB. (**B**) The wild-type N protein, N93A94A (93S and 94D mutated to alanine) mutant protein, N∆93 mutant protein, and GST protein were purified from *E. coli*. Balb/c mice were randomly divided into four groups, with nine mice immunized with N protein and its mutant proteins in each group and five mice immunized with GST protein. Each mouse received an initial immunization of 2 μg of each protein, followed by a booster immunization with an additional 2 μg of each protein 14 days later. Two weeks after the booster immunization, blood was collected from the eyes, and serum was isolated for antibody detection by competitive ELISA (cELISA). (**C**) Growth kinetics of the mutant virus (rGSWW15/N∆93) and the wild-type virus (rGSWW15). MARC-145 cells were infected at an MOI of 0.1 with passage 20 of the mutant virus and the wild-type virus. Cell cultures were collected at different time points after infection and were freeze-thawed three times. Virus infectivity was calculated by 50% end-point titration according to the Karber method. (**D**) MARC-145 cells were mock-infected or infected with PRRSV rGSWW15, rGSWW15/N∆93 and rGSWW15/N93A94A strains at an MOI of 0.1 for 24 h. Immunofluorescent assay was performed using C8 mAb and a commercial anti-PRRSV N antibody SR30.

To identify an appropriate marker site, we conducted an analysis of amino acid conservation within positions 92–103 (Fig. S1). Our investigation revealed that amino acids 92–94, 97–99, and 100–102 exhibited relatively lower conservation levels. Consequently, we proceeded to generate infectious clones harboring three-point mutations at these specific sites. The schematic diagram depicting the construction of infectious clones is presented in Fig. 7A. Regrettably, despite our efforts, we encountered difficulties in rescuing the viruses.

After unsuccessful attempts with the triple and double point mutations, we next evaluated single amino acid deletions. To evaluate the capability of the cELISA in detecting antibodies against the N-mutant protein, we immunized mice with the N93A94A and the N∆93 mutant proteins expressed in *E. coli*. Remarkably, the cELISA was still able to detect antibodies produced by the N93A94A protein but not the N∆93 protein in mouse serum (Fig. 7B). We subsequently rescued the rGSWW15/N∆93 virus, which contains a point deletion at serine 93 of the N protein, derived from a genotype 2 strain of PRRSV. Furthermore, IFA results demonstrated that the rGSWW15/N∆93 recombinant virus showed no reactivity with the C8 antibody (Fig. 7D).

To investigate the impacts of N protein mutations on virus growth, MARC-145 cells were infected in parallel (at an MOI of 0.1) with either rGSWW15 or rGSWW15/N∆93 strains. The multiple-step growth curves demonstrated that the mutant virus replicated with slightly slower kinetics, reaching peak titer approximately 24 h later than the wild-type virus (Fig. 7C). The peak viral titer of the rGSWW15/N∆93 virus was not significantly different from that of the wild-type virus.

### *In vivo* characterization of rGSWW15/N∆93

**Fig 8:**
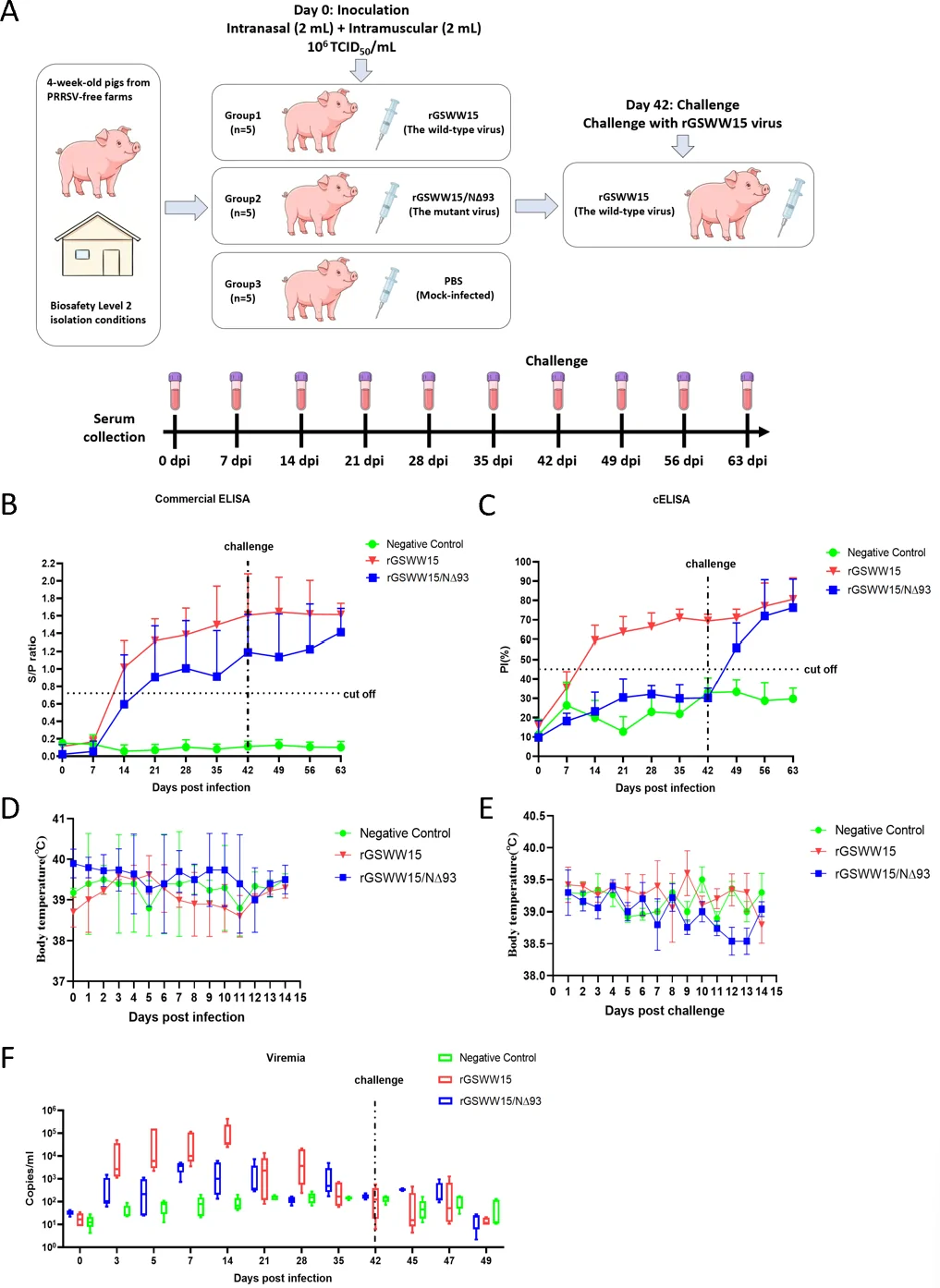
*In vivo* characterization of the mutant marker viruses. (**A**) Schematic illustration of the animal experimental design showing the infection and challenge schedule. Piglets were inoculated with PBS or PRRSV rGSWW15 (the wild-type strain) and rGSWW15/N∆93 (the mutant strain) and challenged with PRRSV rGSWW15 strain at 42 dpi. B and C serum samples collected at 0, 7, 14, 21, 28, 35, 42, 49, 56, and 63 dpi from these pigs were detected by a commercial KQ ELISA kit and our cELISA kit. (**B**) KQ values were expressed according to the KQ ELISA kit [KQ = (Sample OD630nm − Negative control OD630nm)/(Positive control OD630nm − Negative control OD630nm)], and the mean values were obtained from serum samples of five pigs in each group. The horizontal bars represent the standard deviation. A dashed line at 0.7 KQ value corresponds to the threshold value above which samples are considered positive for PRRSV-specific antibodies. (**C**) The values represent the mean PI value from serum samples of five pigs in each group. The horizontal bars show the standard deviation. The dashed line indicates the cut-off point. (**D**) Mean rectal temperatures post-infection. (**E**) Mean rectal temperatures post-challenge. (**F**) Virus titers in the anticoagulated blood of the inoculated and challenged piglets at 0, 3, 5, 7, 14, 21, 28, 35, 42, 45, 47, and 49 dpi were detected by real-time PCR. The values represent the mean number of virus copies from five pigs in each group.

The serological responses to the mutant viruses were evaluated by inoculating three groups of piglets with a total dose of 10^6^ TCID_50_/mL of rGSWW15 and rGSWW15/N∆93 intranasally and intramuscularly (Fig. 8A). The PRRSV-specific antibody response was measured using a commercially available ELISA kit. At 14 days post infection (dpi), sera collected from the wild-type virus-infected pigs were positive for PRRSV-specific antibodies. In the mutant virus-infected group, two pigs seroconverted at 14 dpi, four at 21 dpi, and all at 28 dpi. In contrast, when using cELISA to detect antibodies against the N protein in the sera of these pigs, we observed that pigs inoculated with the mutant virus did not develop antibodies against the epitope. However, strong reactivity was observed in sera from animals infected with the wild-type virus (Fig. 8B and C). More severe clinical signs, including lethargy, emaciation, and anemia, were observed in the group infected with the wild-type virus. The mutant virus group also exhibited mild clinical signs, including coughing and sneezing, but the clinical symptoms were milder than those observed in the wild-type virus group. At 42 dpi, pigs inoculated with rGSWW15 or rGSWW15/N∆93 were challenged with the wild-type rGSWW15 strain. No increase in body temperature was observed in any of the groups (Fig. 8D and E). To determine whether the rGSWW15/N∆93 mutant virus replicated *in vivo*, viremia was determined by real-time PCR (Fig. 8F). Viremia was detectable at 3 dpi with rGSWW15 wild-type virus infection, peaked at 14 dpi, and disappeared at 35 dpi. Similarly, viremia was also detected at 3 dpi with the rGSWW15/N∆93 mutant virus infection, peaked at 7 dpi, and persisted until 35 dpi. Throughout the experiment, the average viral loads of pigs infected with the mutant virus were consistently lower than those of pigs infected with the wild-type virus. To assess the stability of the mutant virus *in vivo*, ORF7 was amplified by RT-PCR from RNA extracted from the whole blood of five infected piglets at 7 dpi. The PCR products were subjected to Sanger sequencing to confirm the presence of the mutation. The results indicated that the mutation remained stably present *in vivo*.

## DISCUSSION

Marker vaccines are essential in programs aimed at controlling or eradicating virus-infected livestock. Incorporating a marker into the vaccine assists in distinguishing between vaccinated animals and those naturally infected with the wild-type virus. PRRS causes tremendous economic losses in the world pig industry. There is a pressing need for a new generation of PRRSV vaccines to enhance protection through immunization. An important direction for future vaccine research is the development of marker vaccines for PRRSV elimination. Currently, there is no DIVA for PRRS control available commercially. Eric Nelson’s group constructed a recombinant green fluorescent protein-tagged PRRSV containing a deletion of an immunogenic epitope, ES4, in the Nsp2 region. Fernando A. Osorio’s group constructed an epitope deletion mutant virus in the Nsp2 (31). Their work provided proof of concept that DIVA PRRSV vaccines could potentially be developed by deletion or insertion of immunodominant epitopes or proteins. Previous studies demonstrated the feasibility of developing a PRRSV-2 DIVA vaccine candidate through N gene modification combined with a companion serological assay (32). In this study, we generated a PRRSV-2 potential negative marker virus by deleting a critical amino acid within an immunodominant epitope of the N protein. The mutant virus retained replication competence while enabling differentiation between vaccinated and naturally infected animals, demonstrating the feasibility of targeting N protein epitopes for DIVA marker development.

The selection of an appropriate genomic region is critical for the development of stable marker vaccines. Previous PRRSV DIVA vaccine studies have mainly focused on Nsp2 due to its high genetic variability and abundance of immunogenic regions suitable for marker modification (10, 33). However, the genetic instability of Nsp2 may compromise marker stability. In contrast, the conserved N protein represents an attractive alternative target. Several antigenic regions within the N protein have been identified, including epitopes located at amino acids 51–67, 69–123, 79–87, 103–109, and 109–113 (34–38). In this study, we identified a novel genotype 2 PRRSV-specific epitope (92LSDSGRISYTVE103) recognized by C8, with serine 93 serving as the critical residue. Deletion of this epitope provides a new approach for developing PRRSV-2 DIVA marker candidates.

To assess whether the point mutation introduced in the mutant virus altered the overall biology of the strain, we evaluated the *in vitro* and *in vivo* phenotype of the epitope deletion mutant. Although rGSWW15/N∆93 exhibited slightly reduced replication capacity and lower viral loads compared with the wild-type strain, it retained comparable pathogenicity in piglets. While attenuation is an essential requirement for live attenuated vaccine development, a single amino acid modification was not expected to confer substantial attenuation. Future studies in our laboratory will focus on identifying additional genetic modifications to generate safer and more effective PRRSV vaccine candidates.

MAbs are widely used as highly specific tools for pathogen detection and therapeutic development. Recent advances in single B cell technology have greatly facilitated the rapid isolation of antigen-specific mAbs and enabled the identification of antibodies targeting conformational epitopes that are difficult to reproduce using conventional immunization and screening approaches. Previous studies have successfully generated PRRSV-specific mAbs using purified viral structural proteins as antigens (39). In this study, we employed purified PRRSV virions as the immunogen source and applied single B cell technology to isolate porcine-derived mAbs targeting native viral antigens. This approach enabled the identification of mAbs recognizing the N protein of genotype 2 PRRSV, providing valuable tools for antigenic characterization and the development of improved diagnostic strategies.

ELISA-based serological assays are essential for the practical application of marker vaccines, as they enable differentiation between vaccinated and naturally infected animals. In this study, we developed a C8-based cELISA that specifically detects antibodies against the conserved N protein epitope absent in the mutant marker virus. The assay effectively discriminated between animals infected with field strains and those vaccinated with rGSWW15/N∆93, highlighting its potential as a companion diagnostic tool for PRRSV-2 DIVA vaccine strategies.

In summary, both *in vitro* and *in vivo* characterization of the rGSWW15/N∆93 revealed that the mutation at serine 93 on the N protein resulted in slower growth kinetics of the mutant virus. In addition, pigs inoculated with rGSWW15/N∆93 did not develop antibodies against the epitope, as measured by the cELISA. However, strong reactivity was observed in sera from animals infected with the wild-type virus. Our study provides evidence supporting the potential application of an immunodominant B-cell epitope deletion within the PRRSV-2 N protein for the development of a DIVA marker virus. Coupling epitope deletion with a companion cELISA represents a promising approach for the development of PRRSV differential diagnostic strategies.

## MATERIALS AND METHODS

### Cells and viruses

Baby hamster kidney cells (BHK-21) were cultured in modified Eagle medium (MEM; Invitrogen) with 10% fetal bovine serum (FBS, Gibco-BRL, MD, USA). MARC-145 cells were cultured in Dulbecco’s minimal essential medium (DMEM; Invitrogen) supplemented with 10% FBS.

The rGSWW15/N∆93 marker virus was constructed from a type 2 PRRSV full-length cDNA infectious clone, GSWW/2015 (40). The marker virus was used for *in vitro* and *in vivo* experiments. The virus titer was determined by 50% tissue culture infective dose per milliliter (TCID₅₀/mL).

### Experimental PRRSV infection of pigs

#### Infection of pigs for single B cell antibody screening

Three PRRSV-negative Landrace pigs and three Juema pigs, a rare pig species well adapted to the harsh environment of the Tibetan Plateau, were used in a sequential heterologous infection study. As described in Table 2, the animals were sequentially inoculated by intranasal or intramuscular injection with different strains of PRRSV. Blood samples were collected on a weekly basis after each vaccination.

**TABLE 2:** Immune procedure of pigs

Immune procedure	Landrace pigs 1498# 1499# 1500#	Juema pigs 0922# 0929# 0968#
First (day 0)	VR2332 vaccine, one dose, intramuscular injection	CH-1R vaccine, one dose, intramuscular injection
Second (day 28)	JXA1-R vaccine, one dose, intramuscular injection	VR2332 vaccine, one dose, intramuscular injection
Third (day 56)	TJM-F92 vaccine, one dose, intramuscular injection	JXA1-R vaccine, one dose, intramuscular injection
Fourth (day 84)	VR2332 10^7^ TCID_50_, nasal drop	TJM-F92 vaccine, one dose, intramuscular injection
Fifth (day 112)	GSWW/2015 10^6.5^ TCID_50_, nasal drop	VR2332 10^7^ TCID_50_, nasal drop
Sixth (day 140)	GSWW/2018 10^6.5^ TCID_50_, nasal drop	GSWW/2015 10^6.5^ TCID_50_, nasal drop
Seventh (day 168)	GSWW/2015 emulsified virions, 10 μg intramuscular injection	GSWW/2018 10^6.5^ TCID_50_, nasal drop
Eighth (day 196)	VR2332 emulsified virions, 10 μg intramuscular injection	GSWW/2015 emulsified virions, 10 μg intramuscular injection
Ninth (day 196)	GSWW/2018 emulsified virions, 10 μg intramuscular injection	VR2332 emulsified virions, 10 μg intramuscular injection

#### Infection of pigs for *in vivo* study of the marker virus

A total of 15 four-week-old pigs were purchased from a PRRSV-free farm. The animals were randomly divided into three groups (*n* = 5 per group). Prior to the start of the experimental inoculations, the animals were housed under Biosafety Level 2 isolation conditions with an acclimation period of 7 days. Two groups were infected intranasally (2 mL) and intramuscularly (2 mL) at day 0 with 10^6^ TCID_50_/mL of the wild-type virus rGSWW15 and the mutant virus rGSWW15/N∆93, respectively, and another group was mock-infected with PBS. Sera were collected from each pig at 0, 7, 14, 21, 28, and 35 dpi. At 42 dpi, rGSWW15 and rGSWW15/N∆93 pigs were challenged with rGSWW15 strain. Antibodies against PRRSV and specific antibodies against the N92-103 epitope were detected by a commercial ELISA kit (KQ) and the cELISA. The inoculated animals were clinically monitored daily. Their rectal temperatures were recorded from day 1 until day 14 post-inoculation.

### Virus neutralization assay

Different PRRSV strains at 200 TCID_50_, diluted in 100 μL of cell culture medium, were incubated with 100 μL of 1:2 serial dilutions of heat-inactivated pig sera in a 96-well plate. Following incubation at 37°C for 1 h, the samples were transferred to a 96-well plate containing a monolayer of MARC-145 cells. The cells were incubated for 5 days and then examined for the cytopathic effect (CPE). The results are reported as the virus neutralization titer, which was determined to be the reciprocal dilution ratio of the sample at which 50% inhibition of the PRRSV-induced CPE was observed.

### Purification of PRRSV virions

PRRSV was propagated on MARC-145 cells. MARC-145 cells were cultured in DMEM medium containing 10% FBS and 1% penicillin and streptomycin at 37°C with 5% CO_2_. When the CPE reached 90%, the cells and supernatant were collected, repeatedly frozen and thawed three times, and centrifuged to eliminate cell debris. The supernatant was concentrated with 7% polyethylene glycol (PEG-6000) and 2% NaCl at 4°C overnight and was separated by centrifugation on 25% to 55% sucrose (in 1× PBS) gradients (38,000 rpm for 4 h at 4°C). Fractions were collected from the top to the bottom of the gradient. A total of 23 fractions (500 μL each) were collected, and each fraction was analyzed by western blot. Fractions containing whole virus antigens were dialyzed with PBS and concentrated after three dialysis steps by centrifugation at 4°C at 4,000 r/min using a 100 kDa ultrafiltration tube.

### Identification of PRRSV-specific plasmablasts in infected pig

EDTA-anticoagulated blood was collected from the precaval veins of pig 0922#, which has a high level of neutralizing antibodies. Blood samples were diluted with PBS, and PBMCs were isolated with HISTOPAQUE 1.077 (Sigma-Aldrich) and then used to identify PRRSV-specific B cells immediately. HP-PRRSV GSWW/2015 strain virions were purified by sucrose gradient centrifugation and biotinylated with EZ-Link NHS-LC-Biotin reagent (Thermo Fisher Scientific, USA). For staining, freshly isolated PBMCs were adjusted to a concentration of 10^6^ cells/200 μL and stained with biotin-PRRSV for 30 min at 4°C in the dark, followed by washing with PBS buffer containing 2 mM EDTA and 0.5% bovine serum albumin (BSA), and then stained with anti-pig IgG FITC, anti-pig IgM PE antibody, and anti-biotin APC antibody for 30 min at 4°C in the dark.

### Single-cell sorting of PRRSV-specific B cells from pig PBMCs using flow cytometry

Pig PBMCs stained as described above were sorted by flow cytometry (BD FACS Aria II, USA). PRRSV-APC^+^/IgG^−^FITC^+^/IgM-PE^−^/events were sorted. Single cells were sorted into 96-well plates (Brand, Germany) containing 10 μL/well of single-cell lysis buffer with 1 μL of DNase (ThermoFisher Scientific, USA). cDNA was synthesized with the SuperScript VILO Master Mix kit (Thermo Fisher Scientific, USA) and carried out as the following program: 25°C for 10 min, 50°C for 10 min, and 85°C for 5 min; the cDNA was stored at −20°C.

### Single-cell PCR amplification and cloning of variable region

Antibody heavy-chain and light-chain variable region genes were amplified by nested PCR using specific primers for pig IgG heavy chain, gamma light chain, and lambda light chain. The first PCR reaction was performed with the outer primer pairs using HotStar Taq Master Mix (Qiagen, Germany) in a total volume of 25 μL per reaction with 2.5 μL cDNA template. The first round of PCR was carried out using the following program: 95°C for 5 min; 95°C for 30 s, 56°C for 50 s, and 72°C for 1 min, followed by 32 cycles; 72°C for 10 min. The second nested PCR was performed with 5 μL of the first-round PCR product as the template in a total volume of 50 μL, and inner primers were used. The final PCR product was visualized on an agarose gel and sent for sequencing. Nested PCR primers used for the amplification of variable region genes of pig IgG were described previously (41).

### Cloning and expression of antibodies

The Ig heavy- and light-chain variable domain sequences were synthesized by GenScript Inc. (www.genscript.com) and cloned into CH-pcDNA3.4 and CL-pcDNA3.4 vectors as described previously (30). To produce recombinant mAbs, the plasmids encoding Ig heavy and light genes were co-transfected into ExpiCHO-S cells (Thermo Scientific) in a ratio of 3:2. After being cultured for 10 days on an orbital shaker platform at 125 rpm with ≥80% relative humidity and 8% CO_2_ at 37°C, the supernatants were harvested and tested for activity. Antibodies were purified as previously described. Briefly, supernatants were loaded onto a HisTrap Talon column, concentrated using a 100 kDa ultrafiltration tube, and then subjected to size-exclusion chromatography (GE Life Sciences) in an AKTA plus protein purification system. The purity and size of porcine mAbs were analyzed by SDS-PAGE. The purified antibodies were stored at −40°C with 20% glycerol.

### Competitive ELISA

cELISA was performed under optimal conditions. Briefly, a 96-well ELISA plate was coated with 1 μg/mL 3A24 at 4°C overnight. The plate was washed three times with PBST (PBS with 0.1% Tween), and then 1 μg/mL 3AN antigen was captured at room temperature for 2 h. Subsequently, the plate was washed three times with PBST and blocked with blocking buffer (5% sucrose and 1% BSA in PBS) at 37°C for 1 h. After being washed three times with PBST, each well was added to 100 μL of mixture containing the optimal dilutions of serum sample and an equal volume of a pretitrated dose (10 ng/mL) of Bio-C8 in PBS and then incubated at 37°C for 1 h. After being washed five times with PBST, 100 μL of 1:30,000-diluted (33 ng/mL) HRP-conjugated streptavidin (GenScript, Inc.) was added, and the plates were incubated at 37°C for 15 min. After being washed five times with PBST, TMB was added (100 μL/well) and incubated at 37°C for 15 min. Finally, 2 M H_2_SO_4_ (100 μL/well) was added to stop the color reaction, and the OD450 nm values were measured using an automatic microplate reader (Bio-Rad, USA).

### Production of 3A24 antibody and 3AN protein

3A24 is a mouse monoclonal antibody that we developed in our lab. It targets a conserved and immunodominant epitope (_109_AEKNPLE_115_) located within the NSP 3A of O/Tibet/CHA/99. It was produced by hybridomas as previously reported. 3AN is a fusion protein consisting of the epitope recognized by 3A24 and the N protein of the PRRSV GSWW2015 strain. It was expressed in *E. coli* and purified with Ni-NTA His-binding resin.

### Indirect immunofluorescence assay

MARC-145 cells were plated in a 24-well plate and inoculated with the PRRSV strain at an MOI of 0.1. At 24 hpi, the cells were fixed with 4% paraformaldehyde solution at room temperature for 15 min and washed with PBS five times. Then the cells were blocked with PBS containing 1% BSA and 0.3% Triton X-100 for 1 h at room temperature and incubated with the indicated antibodies at a working concentration of 5 μg/mL at 4°C overnight. After being washed with PBS five times, cells were incubated with FITC-labeled rabbit anti-porcine IgGs (Thermo Fisher) in the dark for 1 h at 37°C. Cells were washed three times with PBS, and immunofluorescence was observed under the EVOS FL Imaging System (Life Technologies).

### Epitope mapping with overlapping N protein peptides by ELISA

A total of 22 overlapping peptides, which cover the full length of the PRRSV GSWW/2015 N protein sequence, were synthesized by GenScript, Inc. The purity of these peptides was more than 90%, as determined by high-performance liquid chromatography. ELISA plates were coated with 50 μg/mL of the synthesized peptides diluted in carbonate buffer (pH 9.6) and incubated overnight at 4°C. After three washes with PBST, 100 μL/well of purified mAbs diluted to 1 μg/mL in PBST were added and incubated for 1 h at 37°C. Plates were washed again five times, and the HRP-conjugated goat anti-porcine IgG was added to the wells and incubated for 30 min at 37°C. TMB substrate was added after five washes. After incubation for 10 min, H_2_SO_4_ solution was added to stop the reaction. The OD value was measured at 450 nm.

### Epitope mapping with GST-tagged N protein mutations by western blot

Sequences encoding N protein fragments or full-length N protein with deletions or mutations were synthesized by GenScript and cloned into the pGEX-6P-1 vector. The recombinant plasmids were transformed into *E. coli* BL21 (DE3) cells and induced with isopropyl-thiogalactopyranoside at a final concentration of 0.5 mmol/L at 37°C for 6 h. After induction, BL21 cells were centrifuged, and supernatants containing recombinant proteins were analyzed by western blot.

### Construction and rescue of the mutant virus

The procedure for construction and rescue of the wild-type virus and the mutant virus was in accordance with previous work (13). The genome of the PRRSV GSWW/2015 strain was divided into two fragments, GS-A and GS-B, by *Nhe* I locus. The T7 promoter sequence and restriction endonuclease digestion sites were added to the 5′ end of the GS-A fragment. Poly (A) tail was added to the 3′ end of the GS-B fragment. GS-A and GS-B sequences were synthesized by GeneScript. Meanwhile, a linker containing the *Sph* I, *Nhe* I, and *Kpn* I loci was synthesized and inserted into the pBR322 vector. The resulting vector was named pBR323. The GS-A fragment sequence was inserted into the pBR323 vector via the *Sph* I/*Nhe* I site in the linker. The resulting plasmid was designated pGS-A. The GS-B fragment was inserted into the pBR323 vector via the *Nhe* I/*Kpn* I digestion site in the linker. The resulting plasmid was designated pGS-B. Using pGS-B as the template, the N∆93 mutant was generated by a point mutation PCR kit (Vazyme). The primers used were listed in Table 3. To verify the introduced deletion, the PCR-amplified regions were sequenced after construction. The resulting products were named pGS-B-N∆93. pGS-A and pGS-B-N∆93 were digested by *Sph* I and *Nhe* I, and the GS-A fragment was ligated into pGS-B-N∆93 to construct the final mutant full-length clone. The final construct was named pGS-AB-N∆93.

**TABLE 3:** Primers

Primer name	Sequence
∆93F	ACTTGTGCCCTGGATTCAGGGAGGATAAGTTACACTGTG
∆93R	TGAATCCAGGGCACAAGTTCCAGCGCCCTGGTT

The full-length plasmid (pGS-AB-N∆93) was digested with *Acl* I, and linearized DNA was used as the template to generate capped RNA using the mMESSAGE mMACHINE Ultra T7 kit (Ambion) according to the manufacturer’s instructions. The transcribed RNA was purified by phenol/chloroform. BHK21 cells were used for electroporation. The culture supernatant from electroporated cells was collected at 48 h post-electroporation and inoculated onto MARC-145 cells. The cells were then monitored on a daily basis for characteristic CPE.

### Growth kinetics of viruses

MARC-145 cells were infected with mutant and parental viruses at an MOI of 0.1. Cell cultures were collected at 12, 24, 36, 48, 60, 72, 84, and 96 hours post infection (hpi), repeatedly frozen and thawed three times. The virus titer was determined by 50% end-point analysis, and the virus titer was expressed in 50% tissue culture infective dose per milliliter (TCID_50_/mL).

### Statistical analysis

Statistical analysis was performed using GraphPad Prism version 6.0 (GraphPad Software, San Diego, CA, USA). Differences in indicators between groups were assessed using a Student’s *t*-test. A two-tailed *P* value of 0.05 was considered significant.

## Data Availability

The sequences of C8 are presented in the supplemental material. The sequences of 3A24 are available from the corresponding author upon reasonable request.

## SUPPLEMENTAL MATERIAL

Figure S1 jvi.01259-26-s0001.docx Alignment of PRRSV N protein sequence by MegAlign.

ASM does not own the copyrights to Supplemental Material that may be linked to, or accessed through, an article. The authors have granted ASM a non-exclusive, world-wide license to publish the Supplemental Material files. Please contact the corresponding author directly for reuse.
