# Supplementary material for: Generation and characterization of a PRRSV-2 DIVA marker virus by deletion of a novel epitope in the nucleocapsid protein
Source: J Virol. 2026 Sep 24;100(10):e01259-26. doi: 10.1128/jvi.01259-26 (PMC13713002; doi:10.1128/jvi.01259-26)
Supplement: Supplementary file 1 [file jvi.01259-26-s0001.docx]

Supplementary information

**Generation and Characterization of a PRRSV-2 DIVA Marker Virus by Deletion of a Novel Epitope in the Nucleocapsid Protein**

Jiwen Sun^1,2,3#^, Jian Wang^1,3#^, Kun Li^1,3^, Delong Wei^1,3^, Jiaoyang Li^1,3^, Pu Sun^1,3^, Dong Li^1,3^, Pinghua Li^1,3^, Yimei Cao^1,3^, Lijie Tang^2^*, Zengjun Lu^1,3^*, Jing Zhang^1,3^*

1. State Key Laboratory for Animal Disease Control and Prevention, College of Veterinary Medicine, Lanzhou University, Lanzhou Veterinary Research Institute, Chinese Academy of Agricultural Sciences, Lanzhou 730000, China.

2. College of Veterinary Medicine, Northeast Agricultural University, Harbin 150030, China.

3. Gansu Province Research Center for Basic Disciplines of Pathogen Biology, Lanzhou 730046, China.

#These authors contributed equally: Jiwen Sun and Jian Wang

*Correspondence: zhangjing@caas.cn; [luzengjun@caas.cn](mailto:luzengjun@caas.cn); tanglijie@neau.edu.cn

Figure S1.

Figure S1. Alignment of PRRSV N protein sequence by MegAlign.

C8 mAb sequences:

HV1: GLVQPGGSLRLSCVASGFTFSSYIVTWVRQSPGKGLEWLAGTGVGEYALYYRNSVRGRFTLSRDNSQNTAYLQMNSLRVEETGRYFCRRGAAESVDLWGPGVEVVVSS

LV1: QEPAMSVSLGGTVTLTCAFSSGSVTRSHWPSWFQLTPGQPPRTLIVSTDSRPTGVPSRFSGAISGYKAALTITGAQPEDEADYVCGVYFTFTKRPFGGGTHLTVLG
